# Impaired Knowledge of Social Norms in Dementia and Psychiatric Disorders: Validation of the Social Norms Questionnaire–Dutch Version (SNQ-NL)

**DOI:** 10.1177/10731911211008234

**Published:** 2021-04-15

**Authors:** E. van den Berg, J. M. Poos, L. C. Jiskoot, B. Montagne, R. P. C. Kessels, S. Franzen, J. van Hemmen, W. S. Eikelboom, E. G. C. Heijboer, J. de Kriek, A. van der Vlist, F. J. de Jong, J. C. van Swieten, H. Seelaar, J. M. Papma

**Affiliations:** 1 Department of Neurology, Erasmus MC University Medical Center, Rotterdam, the Netherlands; 2 Dementia Research Center, University College London, London, UK; 3 Psychodiagnostic department Eemland, GGZ Centraal Psychiatric Center, Amersfoort, the Netherlands; 4 Experimental Psychology, Helmholtz Institute, Utrecht University, The Netherlands; 5 Donders Institute for Brain, Cognition and Behaviour, Radboud University Nijmegen, the Netherlands; 6 Department of Medical Psychology & Radboud umc Alzheimer Center, Radboud University Medical Center, Nijmegen, the Netherlands; 7 Vincent van Gogh Institute of Psychiatry, Venray, The Netherlands

**Keywords:** social cognition, assessment, Alzheimer’s disease, frontotemporal dementia, reliability

## Abstract

The Social Norms Questionnaire–Dutch version (SNQ-NL) measures the ability to understand and identify social boundaries. We examined the psychometric characteristics of the SNQ-NL and its ability to differentiate between patients with behavioral variant frontotemporal dementia (bvFTD; *n* = 23), Alzheimer’s dementia (AD; *n* = 26), chronic psychiatric disorders (*n* = 27), and control participants (*n* = 92). Between-group differences in the Total score, Break errors, and Overadhere errors were examined and associations with demographic variables and other cognitive functions were explored. Results showed that the SNQ-NL Total Score and Break errors differed between patients with AD and bvFTD, but not between patients with bvFTD and psychiatric disorders. Modest correlations with age, sex, and education were observed. The SNQ-NL Total score and Break errors correlated significantly with emotion recognition and verbal fluency but not with processing speed or mental flexibility. In conclusion, the SNQ-NL has sufficient construct validity and can be used to investigate knowledge of social norms in clinical populations.

Impairment in social behavior, that is the inability to recognize, manipulate, and behave with respect to socially relevant information, is increasingly recognized as a key deficit in neurodegenerative and psychiatric disorders (Kennedy & Adolphs, 2012; Shany-Ur & Rankin, 2011). For example, behavioral variant frontotemporal dementia (bvFTD) is characterized by profound changes in personality, emotions, and behavior, including disinhibition, emotional blunting, indifference, and social disengagement resulting from atrophy of the frontal and temporal lobes (Neary et al., 1998; Rascovsky et al., 2011). In patients with Alzheimer’s dementia (AD) impairments in social behavior are generally less pronounced, but deficits in recognizing emotions (Bediou et al., 2009) and understanding the mental state of others (Castelli et al., 2011) are frequently reported. Also, neuropsychiatric symptoms such as apathy/indifference, loss of empathy, irritability and social withdrawal may be present in over 80% of patients, even in the early stages of the disease (Lyketsos et al., 2011). Several psychiatric disorders, particularly those in the spectrum of schizophrenia/psychotic disorders, are also accompanied by (core) impairments in social behavior (Couture et al., 2006; Fett et al., 2011).

The central processes that are necessary for effective social behavior are referred to as *social cognition* (Adolphs, 2009). Three hierarchical levels of social cognition are recognized, ranging from the perception and automatic attribution of social information (Level 1), understanding and interpreting the personal emotional relevance of social information (Level 2), to higher order processes such as regulating behavioral responses, maintaining and accessing common social knowledge (e.g., norms), and moral decision making (Level 3; Adolphs, 2009; Beauchamp & Anderson, 2010). The perception of social and emotional information (Level 1) and theory of mind (Level 2) are relatively well-studied in neurodegenerative and psychiatric disorders. Examples include deficits in facial emotion recognition in bvFTD and AD (Jiskoot et al., 2020; Torralva et al., 2009; Wiechetek Ostos et al., 2011), impaired (affective and cognitive) theory of mind in schizophrenia (Vucurovic et al., 2020), and reduced empathy in psychotic disorders (Green et al., 2015). However, knowing and understanding social norms (Level 3) has received much less attention. This is surprising as acquired social knowledge, for example, linguistic concepts, behavioral norms, schemas for common social situations, is essential for knowing how to behave in social settings. The Social Norms Questionnaire (SNQ) was developed to determine the degree to which a person understands and can accurately identify implicit, but widely accepted social boundaries (Kramer et al., 2014). A total score and two error scores can be derived from the SNQ, indicating an estimation of knowledge of social norms as well as a tendency to break or to be overly adherent to these norms.

To date, a small number of studies have examined the psychometric characteristics and neuropsychological correlates of the SNQ in patients with dementia. Patients with bvFTD tend to have a lower SNQ Total score than patients with AD and control participants (Fong et al., 2017; Panchal et al., 2016; Possin et al., 2013), but not invariably (Baez et al., 2014). Overadherence appears to be particularly sensitive in discriminating patients with bvFTD from those with AD (Panchal et al., 2016). Only one study examined the SNQ in patients with major psychiatric disorders, showing no significant differences between patient with schizophrenia, bipolar disorder, and control participants (Baez et al., 2013). Whether the SNQ is able to differentiate patients with dementia from patients with psychiatric disorders is yet unknown. In-depth analysis of discriminative ability of the SNQ may aid clinicians in their complex assessment of those reporting cognitive complaints as well as behavioral changes in the memory clinic and psychiatric settings. In healthy participants, the SNQ is correlated with age, but not sex, education, or IQ (Baksh et al., 2018). This is somewhat surprising given previous research showing sex differences in measures of social cognition (e.g., Proverbio et al., 2017). Moreover, the association between the SNQ and other cognitive functions—particularly other measures of social cognition—has not been studied in detail.

The present study aimed to examine (a) the psychometric characteristics of the SNQ (Dutch translation: SNQ-NL); (b) its ability to discriminate between patients with bvFTD, AD, psychiatric disorders, and control participants; and (c) the association between the SNQ and other cognitive functions. Reference data based on control participants are also reported to aid the application of the SNQ-NL in clinical practice.

## Materials and Method

### Participants

This study included 59 patients with bvFTD (*n* = 23) or AD (*n* = 36), who visited the memory clinic of the Alzheimer Center Erasmus MC in Rotterdam and the Radboudumc Alzheimer Center in Nijmegen, the Netherlands, between August 2017 and March 2020 for a standardized work up consisting of a neurological and neuropsychological assessment, laboratory testing (including lumbar puncture in subsample) and structural brain imaging. Clinical diagnoses were made in a multidisciplinary consensus meeting with an experienced neurologist, geriatrician, neuropsychologist, and/or radiologist. Patients with bvFTD met clinical diagnostic criteria for probable bvFTD (Rascovsky et al., 2011). Five patients with bvFTD were part of an ongoing epidemiological study of pathologically confirmed genetic FTD families (Dopper et al., 2014). Patients with AD met the NINCDS-ADRDA criteria for probable AD (McKhann et al., 2011). The group of patients with psychiatric disorders consisted of 27 inpatients with severe chronic psychiatric disorders requiring long-term psychiatric treatment and care at the Zon and Schild location of GGZ Centraal in Amersfoort, the Netherlands. All patients fulfilled a *Diagnostic and Statistical Manual of Mental Disorders–Fifth edition* (*DSM-5*) diagnosis in the schizophrenia/psychotic disorders spectrum (schizophrenia *n* = 23; schizoaffective disorder *n* = 3; delusional disorder *n* = 1) with a prolonged course (>2 years), and impairment in social and/or societal functioning. Control participants (*n* = 92) were community-dwelling older persons recruited through community centers and word of mouth from the greater Rotterdam area (Rotterdam, Schiedam, Barendrecht) and the municipality of Tholen in the Netherlands. Control participants were included when they had no self-reported history of neurological or psychiatric disorders, a depression score <11 on the Hospital Anxiety and Depression Scale (HADS; Bjelland et al., 2002) and a Mini Mental-State Examination (MMSE) score >25. All participants were well-acculturated to the dominant Dutch culture (i.e., had lived in the Netherlands >20 years). An exclusion criterion for all participants was the inability to provide valid answers on the SNQ-NL. When it was unclear whether a participant’s cognitive or behavioral deficits caused them to answer in a stimulus-bound or otherwise meaningless manner the validity of person’s performance was examined by determining the ratio of *Yes* to *No* responses on the SNQ-NL. As proposed by Knopman and Kukull (2015) cases with a *Yes* to *No* ratio <0.3 or >5 were inspected and invalid cases (e.g., response bias unrelated to the content of specific items; 22 *Yes* or *No* answers; no signs of effort to differentiate between the items) were excluded (1 control, 2 AD, 4 bvFTD). Data collection at the Erasmus MC University Medical Center was approved by the institutions’ medical ethics committee. At GGZ Centraal and Radboudumc, data were collected and stored in accordance with the General Data Protection Regulation and the institutions’ ethical guidelines, stored pseudonymously, and analyzed anonymously. All participants gave written informed consent for their data, collected as part of routine neuropsychological assessment, to be used for scientific analysis. We report how we determined our sample size, all data exclusions, all manipulations, and all measures in the study.

### SNQ-NL

The SNQ measures the degree to which a person understands and can accurately identify implicit but widely accepted social boundaries (Kramer et al., 2014). It consists of 22 *Yes*-*No* questions to be completed by the participant after a standardized instruction by the examiner. Examples of included questions are (Would it be socially acceptable and appropriate to . . . ) “Spit on the floor?” (No), “Tell a coworker your age?” (Yes), and “Talk out loud during a movie at the theater?” (No). The total score can be calculated (0 to 22, higher score reflects better knowledge of social norms) as well as the number of errors made in the direction of breaking a social norm (Break errors 0 to 12, higher scores reflect more errors) and the number of errors made in the direction of over adherence to a perceived social norm (Overadhere errors 0 to 10, higher score reflects more errors). As described in the Participants section, the ratio between the number of *Yes* and *No* answers can be used to check for response bias, for example, if a person answers *Yes* or *No* to all 22 items resulting in a meaningless score.

For the development of the authorized SNQ-NL—with permission from K. P. Rankin, who developed the original version of the instrument (Kramer et al., 2014)—both a cultural and a literal translation were performed. As social norms can greatly differ between regions of the world all 22 items were judged by two independent raters (EB, EH) against common social boundaries in the Netherlands, resulting in changing Item 16 “Eat ribs with your fingers” (*Yes*) to “Eat fries with your fingers” (*YES*) as this can be viewed as a Dutch equivalent. The SNQ was translated from English into Dutch by two independent raters (EB, EH). Differences between the translations were resolved by consensus and the resulting consensus translation was then back-translated by a native English speaker. Differences between the original English language version and the back-translated version were examined and minor textual changes were made. In all patient groups, the SNQ-NL was administered as part of the routine neuropsychological assessment.

### Other Measures of Cognition and Mood

All participants performed a neuropsychological assessment, but the test batteries differed between the groups. For the present analysis, we used only those tests that were available for all groups. The MMSE was included as a measure of global cognition (Folstein et al., 1975), the Trail Making Test parts A, B and the B/A index (Corrigan & Hinkeldey, 1987) as measures of information processing speed and mental flexibility, and category and letter fluency (Schmand et al., 2008) as measures of executive functioning. The Emotion Recognition Test (Kessels et al., 2014) was available for the bvFTD group, the psychiatric group and control group and was included as a measure of emotion recognition (social cognition) to examine construct validity of the SNQ-NL. Additional measures included in the present analysis were the HADS (Spinhoven et al., 1997) as a measure of symptoms of anxiety and depression. For the patients with psychiatric disorders, the Screener for Intelligence and Learning Disabilities (Nijman et al., 2018) was used as an indication of mild intellectual disability (MID). Level of education was recorded using seven categories in accordance with the Dutch educational system (1 = less than primary school, 7 = academic degree; Duits & Kessels, 2014), which is comparable with the International Standard Classification of Education (UNESCO, 2011). The estimated years of education for comparison with the Anglo-Saxon educational system are presented in Table 1.

**Table 1.** Characteristics of the Control Participants and the Patient Groups.

	Control	bvFTD	AD	Psychiatric	*p* Value for between group difference^a^
*n*	92	23	36	27	—
Age	62.3 ± 9.4	65.2 ± 8.8	70.1 ± 9.2	49.4 ± 14.4	<.01 (psych < AD = bvFTd = con)
Male sex (%)	47 (51%)	18 (78%)	16 (44%)	20 (74%)	<.01 (bvFTD = psych > con = AD)
Education,^b^ years	12.1 ± 3.0	11.0 ± 3.0	11.1 ± 3.5	10.0 ± 2.5	<.05 (psych < bvFTD = AD = con)
MMSE	28.8 ± 1.3	24.8 ± 5.6	21.9 ± 3.8	24.7 ± 4.8	<.01 (con > bvFTD = psych > AD)
HADS Anxiety subscale	4.1 ± 2.5	5.4 ± 3.4	5.8 ± 3.7	8.0 ± 4.4	<.01 (con < bvFTD = AD < psych)
HADS Depression subscale	2.1 ± 2.1	6.5 ± 4.9	4.5 ± 3.0	5.2 ± 3.3	<.01 (con < bvFTD = AD = psych)
SCIL ≤ 15, *n* (%)	—	—	—	16 (50)	*na*
Months since symptom onset	*na*	49 ± 51	36 ± 29	—	*ns*
*Neuropsychological tests*					
Trail Making A (sec.)	40 ± 17	71 ± 43	92 ± 55	63 ± 50	<.01 (con = psych > bvFTD = AD)
Trail Making B (sec.)	92 ± 55	188 ± 109	265 ± 78	156 ± 70	<.01 (con > psych = FTD > AD)
Trail Making B/A index	2.3 ± 0.6	2.7 ± 0.9	3.5 ± 1.7	2.8 ± 1.2	<.01 (AD < bvFTD = psych = con)
Category fluency (animals)	21.4 ± 5.3	12.7 ± 5.8	12.7 ± 5.7	17.1 ± 5.0	<.01 (con > psych = bvFTD = AD)
Letter fluency (“D-A-T”)	33.5 ± 10.9	19.1 ± 12.8	21.1 ± 11.0	—	<.01 (con > bvFTD = AD)
Emotion Recognition Test (0-96)	52.0 ± 8.6	36.4 ± 6.9	—	42.6 ± 11.2	<.01 (bvFTD < psych < con)

*Note*. Data are means ±*SD* or *n*(%) unless otherwise specified. MMSE = Mini Mental-State Examination; HADS = Hospital Anxiety and Depression Scale; SCIL = Screener for Intelligence and Learning Disabilities; bvFTD = behavioral variant frontotemporal dementia; AD = Alzheimer’s dementia; con = controls; psych = group of patients with psychiatric disorders.

a Adjusted for age, sex, and level of education. ^b^Level of education recorded according to Duits and Kessels (2014; 1 = less than primary school, 7 = university degree) and converted into years of education.

### Statistical Analysis

The statistical approach consisted of three steps. In the first set of analyses, the psychometric characteristics of the SNQ-NL were examined. Internal consistency was assessed with the Kuder–Richardson reliability and the Spearman–Brown split-half reliability analysis. The relation between demographics and the SNQ-NL Total score, Break errors, and Overadhere errors was examined with correlation analysis (Pearson or Spearman where appropriate). Sex differences in the SNQ-NL variables were examined with analysis of variance. In the second step of analysis, between-group differences in the SNQ-NL Total score, Break errors, and Overadhere errors were examined with analysis of covariance adjusted for age, sex, and level of education with subsequent post hoc pairwise comparisons (Bonferroni corrected). These analyses were additionally adjusted for disease duration and severity (months since symptom onset—only available for bvFTD and AD—and MMSE, respectively). Differences between sporadic (*n* = 18) and familial (*n* = 5) bvFTD, and between patients with psychiatric disorders with (*n* = 16) and without (*n* = 16) MID were also explored. In the third step of analysis, exploratory partial correlation analysis (adjusted for age, sex, and education level) between the SNQ-NL variables (Total score, Break errors, and Overadhere errors) and the Trail Making Test, verbal fluency, and the ERT was performed in the patient group as a whole. Provisional reference data were calculated based on the percentile distribution of the SNQ-NL Total score, Break Score, and Overadhere score in the control group. Statistical analyses were performed using SPSS Statistics 25.0 (IBM Corp., Armonk, NY). Previous studies on between-group analysis of the SNQ report medium effect sizes (Cohen’s *d* 0.5 to 0.6; Fong et al., 2017; Panchal et al., 2016; Possin et al., 2013). In line with these prior findings, our sample size is sufficient to detect medium sized effects with a power of 0.8 and α set at .05 (calculation performed with G*Power 3.1; Faul et al., 2009).

## Results

Table 1 shows the characteristics of the participants. The patients with psychiatric disorders were significantly younger than the other groups, *F*(3, 174) = 22.1, *p* < .001, η_p_^2^ = 0.28. In both the bvFTD and the psychiatric group, the proportion of men was significantly higher than in the AD group and the control participants, χ^2^(3) = 11.1, *p* < .05. The psychiatric group had fewer years of education compared with the other groups, *F*(3, 174) = 3.7, *p* < .05, η_p_^2^ = 0.06. The patient groups scored significantly lower than control participants on the MMSE. MMSE score was lowest in the AD group (range 13-29) and comparable between bvFTD (range 11-30) and patients with psychiatric disorders, range 11-30; *F*(3, 167) = 38.2, *p* < .001, η_p_^2^ = 0.41. Time since symptom onset did not differ significantly between bvFTD and AD, *F*(1, 56) = 1.58, *p* = .22, η_p_^2^ = 0.03. The patient groups had mild to moderate Anxiety and Depression subscores on the HADS that were significantly higher than in the control participants, HADS Anxiety *F*(3, 156) = 9.9, *p* < .001, η_p_^2^ = 0.16; HADS Depression *F*(3, 156) = 16.7, *p* < .001, η_p_^2^ = 0.25. Half of the patients in the psychiatric group had MID.

### Psychometric Characteristics of the SNQ-NL

In the total sample (*n* = 178), the SNQ-NL Total score ranged from 7 to 21 with a mean of 17.4 ± 2.6 points. The SNQ-NL Break score ranged from 0 to 11, mean 1.8 ± 1.8. The SNQ-NL Overadhere errors ranged from 0 to 8, mean 2.8 ± 2.0. In the control group (*n* = 92), the SNQ-NL Total score ranged from 14 to 21, mean 18.7 ± 1.6; the SNQ-NL Break score ranged from 0 to 5, mean 1.5 ± 1.1; the SNQ-NL Overadhere errors ranged from 0 to 7, mean 1.9 ± 1.5. There were no floor or ceiling effects on any of the 22 items, although Item 21 (“Talk out loud during a movie at the theater?”) was answered incorrectly by only seven participants (4%). Based on the 22 items of the SNQ-NL in the total sample the Kuder–Richardson coefficient was 0.59 and the Spearman–Brown coefficient was 0.64. Interitem correlations were medium to high (median 0.83, interquartile range 0.72 to 0.92). Removal of the two items with the lowest interitem correlation (Item 4: “Ask a coworker their age?” Item 13: “Keep money you find on the sidewalk?”) increased internal consistency coefficients to 0.65 (Kuder–Richardson coefficient) and 0.70 (Spearman–Brown coefficient).

In the total sample, age showed a small but statistically significant negative correlation with SNQ-NL Total score (*r* = −.16, *p* < .05) and a positive correlation with SNQ-NL Overadhere errors (*r* = .25, *p* < .01). Level of education showed a positive correlation with SNQ-NL Total score (*r* = .24, *p* < .01) and a negative correlation with SNQ-NL Overadhere errors (*r* = −.21, *p* < .01). Age and level of education did not correlate with the SNQ-NL Break score. Men showed a lower SNQ-NL Total score and significantly more SNQ-NL Break and SNQ-NL Overadhere errors than women (Table 2).

**Table 2.** Sex Differences in Performance on the SNQ-NL in the Total Sample.

	Men	Women	Statistic
*n*	101	77	—
SNQ-NL Total score (range 0-22)	16.9 ± 2.7	18.1 ± 2.3	*F*(1, 177) = 9.3, *p* < .01, η_p_^2^ = 0.05
SNQ-NL Break errors (range 0-12)	2.1 ± 1.9	1.5 ± 1.6	*F*(1, 177) = 4.4, *p* < .05, η_p_^2^ = 0.02
SNQ-NL Overadhere errors (range 0-10)	3.0 ± 2.2	2.4 ± 1.8	*F*(1, 177) = 4.2, *p* < .05, η_p_^2^ = 0.02

*Note*. Data are *M* ± *SD*. SNQ-NL = Dutch version of the Social Norms Questionnaire.

### Between-Group Analysis

Age-, sex-, and education-adjusted analysis of variance showed a significant main effect of Group on the SNQ-NL Total score, the Break errors and the Overadhere errors (Table 3). All three patient groups had a lower SNQ-NL Total score compared with the control group, *F*(3, 171) = 17.1, *p* < .001, η_p_^2^ = 0.23. The AD group outperformed the bvFTD and the psychiatric group. There was no significant difference between the patients with bvFTD and those with psychiatric disorders. The bvFTD group and the psychiatric group had more SNQ-NL Break errors compared with the AD group and the control participants, *F*(3, 171) = 4.0, *p* < .05, η_p_^2^ = 0.07. With regard to the SNQ-NL Overadhere errors all three patient groups made more errors than the control participants, *F*(3, 171) = 13.1, *p* < .001, η_p_^2^ = 0.19, but there were no differences between the three patient groups. Post hoc there was no significant difference between patients with sporadic (*n* = 18) versus familial bvFTD (*n* = 5) for the SNQ-NL Total score, *F*(1, 22) = 0.31, *p* = .58, η_p_^2^ = 0.02, Break errors, *F*(1,22) = 0.37, *p* = .55, η_p_^2^ = 0.02, or Overadhere errors, *F*(1, 22) = 1.69, *p* = .21, η_p_^2^ = 0.07. In the psychiatric group, there was no significant difference between patients with (*n* = 12; SNQ-NL Total score 14.9 ± 4.0) and without (*n* = 15; SNQ-NL Total score 16.9 ± 2.4) MID on the SNQ-NL Total score, *F*(1, 26) = 2.6, *p* = .12, η_p_^2^ = 0.09, the number of Break errors, *F*(1, 26) = 1.6, *p* = .22, η_p_^2^ = 0.06, or Overadhere errors, *F*(1, 26) = 0.7, *p* = .40, η_p_^2^ = 0.03. Additional adjustment for time since symptom onset or MMSE score did not notably change the results (data not shown). All between group analyses (as described in the next paragraph) were repeated after removal of SNQ-NL Items 4 and 13, but this did not notably change the results.

**Table 3.** Performance on the SNQ-NL.

	Control	bvFTD	AD	Psychiatric	Statistics	Between group difference^a^
SNQ-NL Total score (range 0-22)	18.7 ± 1.6	15.2 ± 2.8	16.6 ± 2.1	16.0 ± 3.3	*F*(3, 171) = 17.1, *p* < .001, η_p_^2^ = 0.23	con > AD > bvFTD = psych
SNQ-NL Break errors (range 0-12)	1.5 ± 0.2	2.3 ± 0.3	1.5 ± 0.3	3.0 ± 2.8	*F*(3, 171) = 4.0, *p* < .05, η_p_^2^ = 0.07	con = AD < bvFTD = psych
SNQ-NL Overadhere errors (range 0-10)	1.9 ± 1.5	4.4 ± 2.0	3.8 ± 2.1	2.9 ± 2.2	F(3, 171) = 13.1, *p* < .001, η_p_^2^ = 0.19	con < AD = bvFTD = psych

*Note*. Data are *M* ± *SD.* SNQ-NL = Dutch version of the Social Norms Questionnaire; bvFTD = behavioral variant frontotemporal dementia; AD = Alzheimer’s dementia; con = controls; psych = group of patients with psychiatric disorders.

a Adjusted for age, sex, and level of education.

### Associations Between SNQ-NL and Other Cognitive Functions

Table 4 shows the correlations between the SNQ-NL Total score, Break errors, and Overadhere errors and performance on other cognitive tests in the total patient group. The SNQ-NL Total score and the Break errors correlated significantly with the ERT total score (*r* = .54, *p* < .01; *r* = −.55, *p* < .01). There was also a significant correlation between the SNQ-NL Total score and category fluency (*r* = .34, *p* < .01). The association with letter fluency was borderline significant (*r* = .27, *p* = .06). The SNQ-NL Break errors correlated significantly with letter fluency (*r* = −.34, *p* < .05). None of the SNQ-NL variables correlated with the Trail Making Test (Part A, B, B/A ratio). The SNQ-NL Overadhere errors did not correlate with the other cognitive functions (all *p* > .05).

**Table 4.** Associations Between the SNQ-NL and Other Cognitive Functions in the Total Patient Group (*n* = 91).

	SNQ-NL Total score	SNQ-NL Break errors	SNQ-NL Overadhere errors
Trail Making Test Part A	−0.17	0.17	0.05
Trail Making Test Part B	−0.20	0.09	0.15
Trail Making Test B/A ratio	0.01	−0.04	0.03
Category fluency	0.34**	−0.23	−0.21
Letter fluency	0.27^†^	−0.34*	−0.04
Emotion Recognition Test	0.54**	−0.55**	−0.11

*Note*. Data are partial correlation coefficients adjusted for age, sex, and level of education. SNQ-NL = Dutch version of the Social Norms Questionnaire.

† *p* = .06. *p < .05. *p < .01.

### Reference Data for Clinical Practice

Based on the control group (*n* = 92, age range 44-82 years, 47 men), we provide provisional reference data for the SNQ-NL Total score (Table 5), Break errors, and Overadhere errors (Table 6). These data are presented for men and women separately; no significant association between age and education and the SNQ-NL variables were observed in the control group.

**Table 5.** Preliminary Reference Data for the SNQ-NL Total Score Based on the Control Group (*n* = 92).

Percentile	Men	Women
	SNQ-NL Total score	SNQ-NL Total score
2	14	15
5	15	16
10	16	17
15	—	18
20	17	—
30	—	—
40	18	19
50	—	—
60	19	—
70	—	20
80	—	21
85	20	—
≥90	21	—

*Note*. SNQ-NL = Dutch version of the Social Norms Questionnaire.

**Table 6.** Preliminary 5th Percentile Cut-Off Scores for the SNQ-NL Break and Overadhere Errors Based on the Control Group (*n* = 92).

Percentile	Men		Women	
	SNQ-NL Break errors	SNQ-NL Overadhere errors	SNQ-NL Break errors	SNQ-NL Overadhere errors
5	4	5	3	4

*Note*. SNQ-NL = Dutch version of the Social Norms Questionnaire.

## Discussion

The present study examined the psychometric characteristics of the SNQ-NL and its ability to discriminate between patients with (subtypes of) dementia, psychiatric disorders, and control participants. The main results show that the SNQ-NL Total score (modestly) correlates with age and level of education, and that women obtained higher SNQ-NL Total scores and made less Break errors than men. The patients with bvFTD, AD or psychiatric disorders performed worse on the SNQ-NL Total Score than control participants. The SNQ-NL was able to differentiate patients with AD from patients with bvFTD and patients with psychiatric disorders (SNQ-NL Total score and Break errors), but there was no difference between patients with bvFTD and the psychiatric group (SNQ-NL Total score and Break errors). Patients made more Overadhere errors than control participants, but there were no differences in Overadhere errors between the individual patient groups. The SNQ-NL Total score and Break errors correlated with emotion recognition and verbal fluency, but not with processing speed or mental flexibility.

Our results on the psychometric characteristics of the SNQ-NL are in line with (scarce) previous reports (Baksh et al., 2018, *n* = 91; Kramer et al., 2014, *n* = 122) indicating a modest negative correlation with age. In contrast to these previous studies, the SNQ-NL Total score and Break errors showed a small, but statistically significant difference between men and women in our analysis. This finding is consistent with previous reports on sex differences on measures of social cognition (e.g., Montagne et al., 2005; Proverbio et al., 2017) and with an observational study on the SNQ in a population-based sample of persons >65 years old (*n* = 744; Ganguli et al., 2018). The internal consistency indices for the SNQ-NL Total score (22 items) were in the 0.6 to 0.65 range, which is considered a marginal reliability, albeit not uncommon for neuropsychological tests or questionnaires (Strauss et al., 2006). Removal of two items with the lowest interitem correlation increased the internal consistency to an adequate level (.70), but still well below the preferable .80 level. Possibly, “social norms” as measured with the SNQ-NL is not a singular concept, but multidimensional in nature. For example, there may be a conceptual difference between items that measure direct social interaction (e.g. “Tell a stranger you don’t like their hairstyle) and items that have a more indirect social consequence (e.g. “Wear the same shirt every day”). Dissociations of this nature have been reported previously, for example, by Rankin et al. (2003) showing a dissociation between (loss of) social dominance and (loss of) social warmth/agreeableness in bvFTD—which was related to predominant temporal versus frontal lobe atrophy in these patients. The size of our control group (*n* = 92) did not allow for an in-depth factor analysis, but future studies in larger samples could provide more insight in the underlying factor structure of the SNQ and the SNQ-NL. Depending on the variable communalities, the variable-to-factor ratio and the dichotomization threshold a minimum sample size of 320 is recommended (Pearson & Mundform, 2010).

With regard to the between-group analysis, the SNQ-NL (Total score and Break errors) clearly differentiated between patients and control participants and between patients with AD and bvFTD. This result has been consistently reported in previous studies as well (Fong et al., 2017; Panchal et al., 2016; Possin et al., 2013). Panchal et al. (2016) for instance reported that—besides the Total score—the number of Overadhere errors differs significantly between patients with AD and bvFTD, possibly resulting from difficulty in recognizing a change in the context of social rules in patients with bvFTD. In our analysis, Break errors proved to be more sensitive than Overadhere errors in discriminating AD from bvFTD which could be associated with greater social/person-based disinhibition in bvFTD compared with AD (Paholpak et al., 2016), leading to increased social rule breaking. Our results on the SNQ-NL extend existing literature on social cognition in bvFTD and AD showing deficits for both patient groups compared with control participants, but worse SNQ-NL performance in patients with bvFTD compared with AD. Differences in the nature and extent of impaired social cognition have been reported for emotion recognition and ToM as well. Impaired facial emotion recognition is viewed as a core feature of bvFTD, related to gray matter loss in the (right) lateral orbitofrontal cortex (Goodkind et al., 2012). In contrast, decreased emotion recognition in AD may reflect a more general cognitive/perceptual impairment (Phillips et al., 2010). Similarly, patients with bvFTD show impairment in most ToM tasks—such as false belief, ToM cartoons or stories, and faux-pas comprehension—which appears to be independent of executive impairments (Adenzato et al., 2010). Patients with AD on the other hand have intact performance on simple false belief tasks, but perform poorly on more complex ToM tasks which is partly related to memory and executive impairment (Castelli et al., 2011). These divergent profiles of impaired social cognition in bvFTD and AD indicate that deficits in social and emotional behavior may depend on other cognitive operations and brain structures.

No previous studies directly compared patients with dementia and patients with psychiatric disorders. Patients with psychiatric disorders had lower SNQ-NL scores than patients with AD, but the SNQ-NL was not able to discriminate between patients with psychiatric disorders and bvFTD. Apparently, the ability to understand and identify social norms was similarly impaired in these groups. Baez et al. (2013) investigated the SNQ in outpatients with schizophrenia (*n* = 15) or bipolar disorder (*n* = 15) who were clinically stable. Baez et al. (2013) showed no differences in Break errors or Overadhere errors compared with control participants, indicating less severely impaired knowledge of social norms in these milder patients. However, the current study included inpatients patients with a severe and prolonged disorder in the schizophrenia/psychotic disorders spectrum that greatly affected social and societal functioning in these patients.

Three previous studies examined the neuropsychological correlates of the SNQ. Baez et al. (2013) did not find any significant correlations between the SNQ Total score and measures of executive functions (inhibitory control, working memory) and social cognition (emotion recognition, social perception) in a group of patients with psychiatric disorders (schizophrenia *n* = 15, bipolar disorder *n* = 15). In contrast, in a sample of patient with dementia (bvFTD *n* = 15, AD *n* = 18). Panchal et al. (2016) showed that the SNQ Total score correlated with executive functions (concept shifting), semantic memory, and verbal fluency. Overadhere errors also correlated with executive functioning and (design) fluency. Break errors did not correlate with other cognitive functions. Similarly, in a population-based sample of persons >65 years old (*n* = 744), Ganguli et al. (2018) showed association between the Total score and Overadhere errors and measures of executive functioning, language, and memory, but Break errors—which were rare in their population based sample—did not show any significant associations with other cognitive functions. In line with Panchal et al. (2016), our results also showed a correlation between the SNQ-NL (Total score and Break score) and verbal fluency. We did not find any consistent correlation with executive functioning, which is possibly due to differences in the executive tasks that were included (Trail Making test in the present study vs. Wisconsin Card Sorting Test in Panchal et al., 2016). However, the lack of association with executive functioning in our analysis could also suggest that although social cognition and executive functioning may involve similar processes, they are also clearly dissociable functions (Lough et al., 2001). Moreover, the correlation between the SNQ-NL (Total score and Break errors) and the measure of facial emotion recognition corroborates the construct validity of the SNQ-NL.

Strengths of the present study include the large study sample and the direct comparison between patients with dementia (AD and bvFTD), patients with psychiatric disorders and control participants. A limitation that needs to be considered is the heterogeneity of the study sample, both across and within the groups. As is true for all studies comparing patients with AD and bvFTD, it is difficult to properly match these groups on disease severity and disease duration. AD and bvFTD generally differ in the age of onset and the course of the disease (e.g., younger onset, more rapid decline in bvFTD than in AD; Roberson et al., 2005). Patients with AD and bvFTD who have a similar age and time since symptom onset may thus differ in disease severity (or the other way around). Moreover, the MMSE is a valid marker of disease severity in AD but not in bvFTD (Premi et al., 2016).

The psychiatric group included patients with a disorder in the schizophrenia/psychotic disorders spectrum who had a prolonged disease course which severely affected social and societal functioning. Other psychiatric disorders may also be accompanied with (core) impairments in social behavior, such as autism spectrum disorders (Barak & Feng, 2016), bipolar disorder (Solé et al., 2011) and mood disorders (Kupferberg et al., 2016). Despite heterogeneity in symptomatology, impairments in social and societal functioning may be similar in size and severity across diagnoses (Hendryx et al., 2009), and considerable overlap in deficits in social cognition have been reported (Bora & Pantelis, 2016). A direct comparison of patients with dementia and psychiatric disorders is also hampered by inherent differences in the course and duration of the diseases that can only partly be adjusted for in statistical analysis. Despite the heterogeneity, we feel that the direct comparison of social cognition in patients with dementia versus psychiatric disorders in our study is a strength, as it is of great importance to understand the similarities and differences in the nature and severity of deficits in social cognition between these disorders. Moreover, insight into the ability of specific tests of social cognition to differentiate between dementia and psychiatric disorders has direct clinical relevance in memory clinics and psychiatric settings. Another limitation of the present study was the small number of different neuropsychological tests that were available for the correlation analysis. In particular, no measure of response inhibition (e.g., Stroop test, Go/No go, informant-based ratings of behavioral disinhibition) was available, preventing inferences on the potential association between inhibitory control and social norms adherence. The only other available measure of social cognition was the emotion recognition as a measure of emotion recognition. Obviously, emotion recognition differs from understanding social norms and additional studies on correlations with other measures of social cognition—both within the level of knowledge/regulation and between measures of emotion perception and ToM—are needed. Moreover, speech comprehension was not assessed in our study sample. In all, our results indicated sufficient construct validity, but these results are preliminary and a more in-depth exploration of the association between the SNQ-NL and other cognitive functions will provide more insight into the processes involved in performance on the SNQ-NL.

The development of the Dutch version expands the number of available translations of the SNQ (French, Spanish, Portuguese, Hebrew, and Canadian English; K. P. Rankin, personal communication). In these translations, both a literal and a cultural translation was performed as social boundaries vary greatly by region of the world. Whether these efforts resulted in equivalent versions of the SNQ remains to be evaluated. The influence of cultural factors on neurocognitive performance is particularly apparent in the assessment of social cognition as is illustrated by a recent study by Quesque et al. (2020) in which social cognition was measured with standard tests in 587 participants from 12 different countries. After controlling for age, sex, and education, differences between countries accounted for over 20% of variance in emotion recognition and theory of mind, which could not be attributed to differences in linguistic variables.

The importance of including measures of social cognition as part of standard neuropsychological assessment in the memory clinic and psychiatric settings is increasingly recognized (Buhl et al., 2013). The number of available psychometrically solid tests for the assessment of social cognition in clinical practice is, however, limited. For the more basic processes of social cognition—that is, perception (Level 1) and theory of mind (Level 2)—some standardized tests are available, such as the Ekman faces (Ekman & Friesen, 1976), Emotion Recognition Test (Kessels et al., 2014), The Awareness of Social Inference Test (McDonald et al., 2006), Happé cartoons (Happé et al., 1999), or Faux Pas Test (Stone et al., 1998). Tests for higher order processes of social cognition—including regulating behavioral responses, maintaining and accessing common social knowledge (e.g., norms), and moral decision making—are much more scarce. This may in part be a matter of time, but it also reflects difficulty in defining and operationalizing the specific neuropsychological processes underlying the theoretical concept of social cognition as a whole and higher order social cognition in particular. An approach in which different social cognitive abilities are comprehensively assessed, such as the Edinburgh Social Cognition Test (Baksh et al., 2018; including cognitive ToM, affective ToM, interpersonal and intrapersonal understanding of social norms and interactions) is also valuable. Investigation of different aspects of social cognition (either at the level of emotion perception, theory of mind, or knowledge and regulation of social behavior) provides an important opportunity in the differentiation between dementia subtypes and psychiatric disorders. As a measure of the ability to understand and identify implicit social norms, the SNQ-NL is a valuable addition to the available instruments. The SNQ-NL may be used by clinicians in both memory clinics and psychiatric settings to (a) help differentiate between neurodegenerative and psychiatric causes of behavioral changes and (b) better understand the underpinnings of difficulty in social and societal functioning experienced by patients (for review and practical guidelines on assessment of social cognition, see Henry et al., 2016). Based on the control group, this study provides preliminary reference data (Table 5; *n* = 92, age range 44 to 82 years, 47 men), which need further validation in larger samples with a wider age range, but aid current researchers and clinicians in the interpretation of a performance on the SNQ-NL, albeit with some caution.

In sum, the SNQ-NL differentiates between patients with AD and bvFTD, but not between bvFTD and psychiatric disorders. The SNQ-NL has sufficient construct validity and can be used to investigate knowledge and understanding of social norms in clinical populations.
